# Roxadustat (FG-4592) abated lipopolysaccharides-induced depressive-like symptoms *via* PI3K signaling

**DOI:** 10.3389/fnmol.2023.1048985

**Published:** 2023-03-15

**Authors:** Axiang Li, Zizhen Liu, Tahir Ali, Ruyan Gao, Yanhua Luo, Qichao Gong, Chenyou Zheng, Weifen Li, Hongling Guo, Xinshe Liu, Shupeng Li, Tao Li

**Affiliations:** ^1^College of Forensic Medicine, Xi’an Jiaotong University Health Science Center, Xi’an, Shaanxi, China; ^2^Institute of Forensic Injury, Institute of Forensic Bio-Evidence, Western China Science and Technology Innovation Harbor, Xi’an Jiaotong University, Xi’an, Shaanxi, China; ^3^State Key Laboratory of Oncogenomics, School of Chemical Biology and Biotechnology, Peking University Shenzhen Graduate School, Shenzhen, China; ^4^Shenzhen Bay Laboratory, Institute of Chemical Biology, Shenzhen, China; ^5^NHC Key Laboratory of Forensic Science, College of Forensic Medicine, Xi’an Jiaotong University, Xi’an, Shaanxi, China; ^6^Department of Psychiatry, University of Toronto, Toronto, ON, Canada

**Keywords:** lipopolysaccharides, depression, Neuroinflammation, HIF-PHD/PI3K, roxadustat (FG-4592)

## Abstract

**Background:**

Despite its role in inflammation and the redox system under hypoxia, the effects and molecular mechanisms of hypoxia-inducible factor (HIF) in neuroinflammation-associated depression are poorly explored. Furthermore, Prolyl hydroxylase domain-containing proteins (PHDs) regulate HIF-1; however, whether and how PHDs regulate depressive-like behaviors under Lipopolysaccharides (LPS)-induced stress conditions remain covered.

**Methods:**

To highlight the roles and underlying mechanisms of PHDs-HIF-1 in depression, we employed behavioral, pharmacological, and biochemical analyses using the LPS-induced depression model.

**Results:**

Lipopolysaccharides treatment induced depressive-like behaviors, as we found, increased immobility and decreased sucrose preference in the mice. Concurrently, we examined increased cytokine levels, HIF-1 expression, mRNA levels of PHD1/PHD2, and neuroinflammation upon LPS administration, which Roxadustat reduced. Furthermore, the PI3K inhibitor wortmannin reversed Roxadustat-induced changes. Additionally, Roxadustat treatment attenuated LPS-induced synaptic impairment and improved spine numbers, ameliorated by wortmannin.

**Conclusion:**

Lipopolysaccharides-dysregulates HIF-PHDs signaling may contribute to neuroinflammation-coincides depression *via* PI3K signaling.

## Introduction

Major depression disorder (MDD) is a global concern with increasing prevalence, expected to be a single leading cause among all disease burdens by 2030. Clinically, MMD is characterized by a lack of energy, persistent low mood, despairs, sleep disorders, and in severe cases, suicidal behaviors (Athira et al., 2020; Gutiérrez-Rojas et al., 2020; Abdoli et al., 2022). Unfortunately, due to its complexity and heterogeneity as determined by genetic and environmental factors, the molecular mechanisms of MDD are enigmatic (Athira et al., 2020). The current hypothesis of depression focuses on the hypothalamus-pituitary–adrenal (HPA) axis, neuroplasticity, and monoamine neurotransmitter depletion (Brigitta, 2002; Hasler, 2010). However, the delayed effect of available antidepressants and lack of focus on cells other than neurons demonstrate the limitation of these specific pathological mechanisms (Andrade and Rao, 2010; Malhi et al., 2020). Thus， around one-third of MDD patients do not respond well to the existing treatments that demand the revitalization of psychiatric therapeutics with novel intervention options that engage non-monoaminergic molecular targets. In recent years, the inflammatory hypothesis has been proposed for depressive symptoms (Madeeh Hashmi et al., 2013; Gałecki and Talarowska, 2018) as an activated immune system has been founded in MMD patients (Cassano et al., 2017; Zou et al., 2018), which opened new avenues for depression investigation. We reported previously that lipopolysaccharides (LPS) could induce neuroinflammation and depressive-like behavior in mice. Besides, it modulated inflammatory signaling molecules, including NLRP3, NF-кB, p-p38, etc., accompanied by depressive symptoms such as immobility, decreased sucrose preferences in the mice, and synaptic defects (Ali et al., 2020b; Li et al., 2021b).

Mechanistically, LPS administration dysregulates PI3K/Akt/NF-kB signaling, which may lead to neuroinflammation. Concurrently, the altered neuroinflammatory response can coincide with synaptic defect *via* different signaling, including HDAC1 and Sirt3/HO-1 signaling (Li et al., 2019; Zhao et al., 2019; Ali et al., 2020a; Jamali-Raeufy et al., 2021; Li et al., 2021b). Similarly, LPS can initiate the transcription factor HIF-1 signaling, which may be mediated through the NF-kB signaling (Frede et al., 2006; Palladino et al., 2018), as NF-kB has a binding site on the promoter of the HIF-1 gene as a transcription factor (van Uden et al., 2008). However, it has also been reported that hypoxia-activated HIF-1 is synergistic with LPS in macrophages (Mi et al., 2008). Similarly, PI3K signaling could contribute to the translation of HIF-1 *via* mTOR (Xu et al., 2020), while its role in neuroinflammation and synaptic defects is not highlighted yet.

Roxadustat (FG-4592) is the reversible inhibitor of hypoxia-inducible factor prolyl hydroxylase (PHDs), a hypoxia-inducible factor (HIF-1) stabilizer, orally available and approved by the FDA. Initially, this drug was approved for treating anemia, including in China. FG-4592 showed considerable protection against other hypoxia-related disorders, including cancer, fibrosis, and chronic inflammation. Besides, in the sepsis animal model, Roxadustat-treatment significantly reduced the cytokines, including IL-1β, IL-6, and TNF-α (Akizawa et al., 2019; Liu et al., 2021; Zhu et al., 2022). Furthermore, prolyl hydroxylases (PHDs) regulate HIF-1 under different conditions (Appelhoff et al., 2004), whose underlying mechanisms have not been explored in LPS-induced stress conditions. Similarly, the HIF-1 anti-inflammatory consensus has not been currently elucidated (Zhu et al., 2022). Therefore, we aimed to determine whether LPS-altered HIF-1 signaling can affect depressive symptoms coinciding with neuroinflammation and synaptic defects. Surprisingly, our data showed that LPS lead to HIF-1 signaling impairment, neuroinflammation, synaptic defects, spine number modification, and depressive symptom, which PI3K-signaling could alter.

## Materials and methods

### Animals

C57BL/6 J male mice (6–8 weeks) were obtained from the Guangdong Medical Laboratory Animal Centre, China. The experimental animals were housed at the Laboratory Animal Research Centre, Peking University Shenzhen Graduate School, under a 12 h light/12 h dark cycle at 18–22°C, *ad libitum* access to food and water throughout the study. The experimental procedures were set in such a way as to minimize mice suffering. The Animal Care and Use Committee of the Experimental Animal Center at Peking University, Shenzhen Graduate School, approved the animal experiments.

Lipopolysaccharides-induced depression is associated with inflammation (Ali et al., 2020a), and FG-4592 has shown anti-inflammatory action by reducing cytokine levels in the animal model (Akizawa et al., 2019; Liu et al., 2021; Zhu et al., 2022). Besides, the HIF-1 anti-inflammatory mechanism is largely unknown. Therefore, this was one reason for HIF-1 stabilizer selection for the LPS-induced neuroinflammation-coinciding depression. The rationale for the FG-4592 treatment for the depression model is unavailable; thus, we initially checked the dose-dependent action of the FG-4592 while performing OFT and SPT tests (Supplementary Figure S1A).

**Figure 1:**
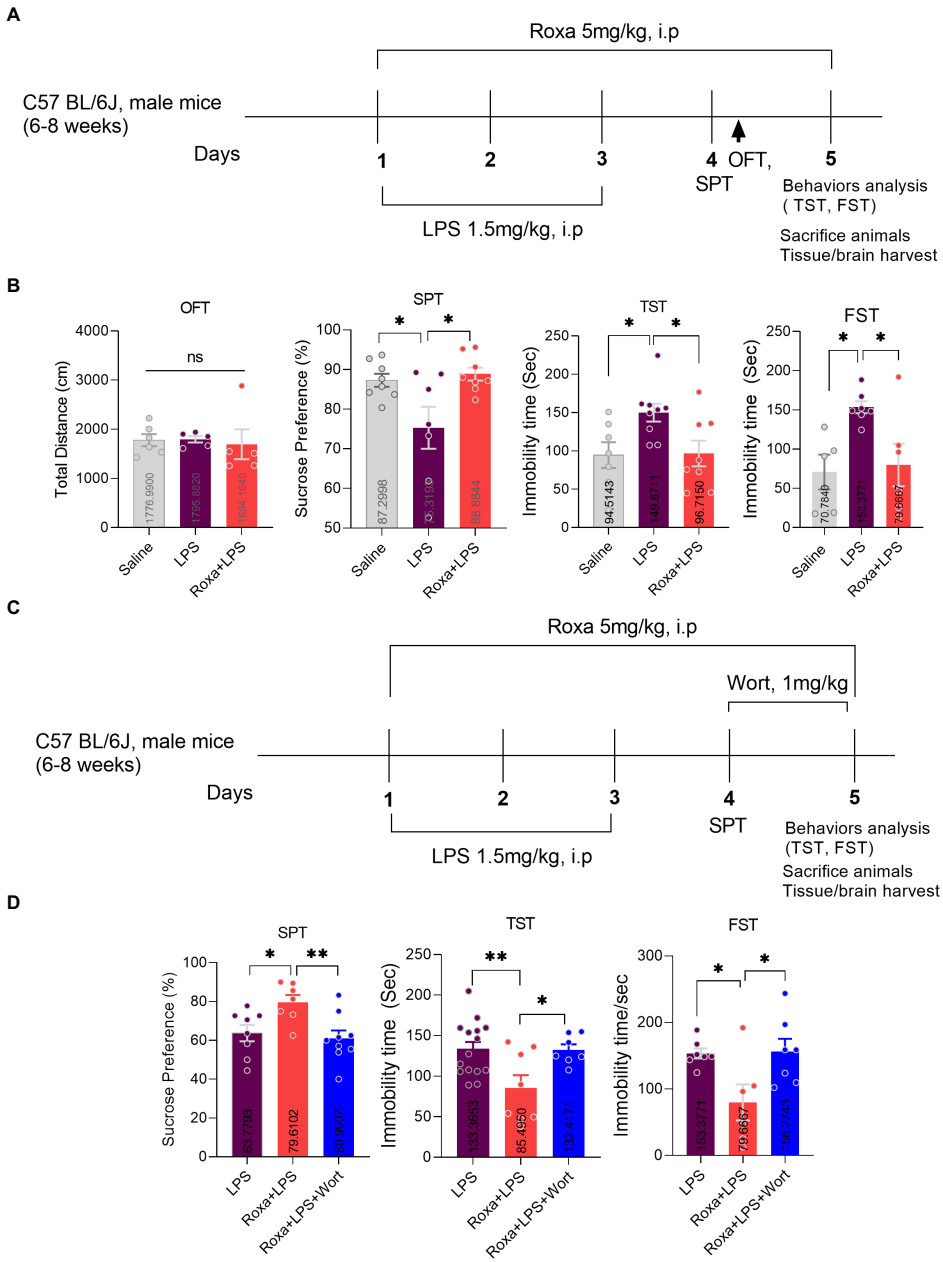
Roxadustat treatment reduced LPS-induced depressive-like behaviors *via* PI3K signaling. **(A)** Drugs treatment schedule, **(B)** open field test (OFT), forced swimming test (FST), and a sucrose preference test (SPT). **(C)** Drugs treatment schedule, **(D)** Sucrose preference Test (SPT), and tail suspension Test (TST). All the values are expressed as mean ± SEM: ANOVA followed by *post hoc* analysis. *p* = <0.05 were considered significant *p < 0.05, **p < 0.01. ns: non-significant.

We performed the present study in two experiments. In the first experiment, animals were assigned to three groups (each group = 6–10): saline-treated group (Saline), LPS-treated group (1.5 mg/kg/day), Roxadustat: FG-4592 (Roxa; 5 mg/kg/day) plus LPS group (Roxa+LPS). LPS (0.1 ml/10 g mice) and Roxadustat (0.1 ml/10 g mice, were intraperitoneally administered to the mice. Besides, Roxadustat was treated 1 h before LPS administration. The drug treatment schedule is shown in Figure 1A. After 24 h of the final LPS administration, behavioral tests were performed. Finally, the mice were sacrificed, and tissues were collected and quickly stored at-80°C until further use. Notably, LPS (Sigma-Aldrich, #L2880) was dissolved with sterile water directly to its working concentration. Roxadustat was dissolved in 5% dimethyl-sulfoxide (DMSO) and was administrated 2 h before the behavior test.

In the second experiment, animals were again divided into three groups (8–15 mice/group): LPS group, Roxa+LPS group, and Roxa+LPS + Wort group (Wortmannin,1 mg/kg). Wortmannin (wort) was administered intraperitoneally for 2 days, before 2 h behavior tests (Figure 1C). Wortmannin was dissolved in 5% DMSO. The behavior and organ/tissues collection process were the same as above.

### Open field test

Open field test (OFT) was performed according to previous protocols (Ali et al., 2020b). Briefly, mice were adapted to the experimental room for 1 h and then placed in a 45 × 45 × 30 cm chamber. Each mouse was started in the center of the chamber and was allowed to move freely for 5 min. The total distance traveled was recorded and analyzed using Smart v3.0 software.

### Sucrose preference test

A sucrose preference test was performed using a two-bottle free-choice paradigm (Liu et al., 2018). Initially, the mice were habituated for 48 h hrs with two drinking bottles (one containing 1% sucrose and the other water) in their home cage. Next, on the third day of the drug administration, the mice were deprived of water and food for 24 h. The next day, each mouse had free access to two bottles of either 1% sucrose solution or standard drinking water. The positions of the bottles were switched halfway through testing to prevent the possible effects of side preference on drinking behavior. Percentage preference for sucrose was calculated at the end of the test using the following formula: Sucrose Preference = Sucrose consumption/ (Sucrose consumption + Water consumption) × 100%.

### Tail suspension test

The tail suspension test was performed as described previously (Li et al., 2022). Mice were suspended by the tails to a rod about 45 cm above the floor by placing adhesive tape 1 cm from the tail tip. By the commonly accepted criteria, immobility was defined as the absence of movement of the animals’ heads and bodies. The immobility time was recorded for 6 min in a dark room. Etho Vision XT software was used for TST recording and analysis.

### Forced swimming test

The forced swimming test (FST) was performed as described previously. Animals were forced to swim in a Plexiglas cylinder (height: 70 cm, diameter: 30 cm) filled with water (23 ± 1°C) to a height of 30 cm. The video was recorded for 6 min, and the immobility time was analyzed during the last 5 min of the test. Mice were immobile when they remained motionless or only made movements necessary to keep their heads above the water’s surface.

### ELISA

According to the manufacturer’s protocols, cytokines expression was quantified using ELISA kits (Elabscience). Briefly, a 100 μL standard or sample was added to the wells and incubated for 90 min at 37°C. The plates were then washed, and a Biotinylated Detection Ab working solution was added to each well. The plates were incubated for 1 h at 37°C. Next, 100ul HRP conjugate working solution was added for 30 min at 37°C. Finally, the reaction was stopped, and the optical density was measured.

### Immunofluorescence

Immunofluorescence staining was performed according to described previously (Li et al., 2022). Briefly, mice were perfusion-fixed with 4% paraformaldehyde and soaked in PFA for 48 h, then brian was replaced with sucrose, and 30um brain tissue sections were prepared. Sections were washed with PBS for 15 min (5 min × 3). After washing, the sections were blocked with blocking buffer (10% normal goat serum in 0.3% Triton X-100 in PBS) for 1 h at room temperature and then incubated with primary antibodies overnight at 4°C, Rabbit anti-Iba1 (1: 1000, Wako,#019–19,741); Mouse anti-GFAP (1,1,000, Sigma-Aldrich, #MAB360). The next day, sections were incubated with secondary antibodies (Alexa Flour secondary antibodies, Thermo Fisher) at room temperature for 1 h; the hoechst was applied in the last 10 min. The sections were washed with PBS for 15 min (5 min × 3). After washing, sections were transferred onto glass slides, and glass coverslips were mounted using a mounting medium. Images were captured using a confocal microscope and were analyzed by ImageJ software.

### Golgi staining

Golgi staining was performed according to Sami Zaqout’s protocols (Li et al., 2021a). Impregnation step: the mice brain sample was kept in Golgi-Cox solution at room temperature in the dark; after 24 h, the sample was transferred into a new Golgi solution-containing bottle with the help of a histological cassette and kept settling at room temperature in the dark for 7–10 days. Tissue protection step: the brain sample is transferred from the Golgi-Cox impregnation solution to a new bottle with the tissue-protectant solution and kept at 4°C in the dark. After 24 h, the tissue-protectant solution is replaced by a new solution in a new bottle for 4–7 days. Sectioning step: the brain sample is embedded in 4% low melting point agarose. 150–200 um sections were prepared using a sliding microtome and mounted to gelatin-coated microscope slides. Then, the brain tissue was placed in a staining solution for 10 min and rinsed with double distilled water, followed by dehydration (sequential rinse 50,75, and 95% ethanol) and xylene treatment finally, examined under an inverted fluorescence microscope IX73 Olympus.

### Real-time quantitative RT-PCR

Total RNA was isolated from the hippocampus with Trizol (Invitrogen, Germany) and reversed to cDNA using the Reverse Transcription System (Promega). Primers ordered with the following sequences: HIF-1, 5′-GAAACGACCACTGCTAAGGCA-3′(forward) and 5′-GGCAGACAGCTTAAGGCTCCT-3′(reverse); prolyl hydroxylase (PHD)1, 5′-GGCCAGTGGTAGCCAACATC-3′(forward) and 5′-GTGGCATAGGCTGGCTTCAC-3′(reverse); PHD2, 5′- TGACCACACCTCTCCAGCAA-3′ (forward) and 5′- CTGCCAACAATGCCAAACAG-3′ (reverse); and PHD3, 5′-GGTGGCTTGCTATCCAGGAA-3′(forward) and 5′-ATACAGCGGCCATCACCATT-3′(reverse).

### Western blotting

Western blotting was also performed according to the standard protocol. Briefly, the protein sample was denatured by boiling at 95°C for 5 min and separated *via* SDS-PAGE. The separated protein was then transferred onto a nitrocellulose membrane. The membrane was blocked with non-fat milk in TBST (Tris-buffered saline, 0.1% Tween 20) and then incubated with primary antibody (1:1000) (Table 1) overnight at 4°C. The next day, the membranes were incubated with a secondary antibody (1:10000) for 1 h at room temperature. For detection, the ECL Super signal chemiluminescence kit was used according to the manufacturer’s protocol. Blots were developed using ChemiDoc MP BIO-RAD. Densitometric analysis of the bands was performed using the Image Lab software. The stripping buffer was purchased from Thermo Scientific (LOT: WE32238 Scientific) blot stripping. Briefly, blots were washed with TBST 3 times. Immerse blot in stripping buffer, followed by incubating for 15-30 min at room temperature. Finally, removed the stripping buffer by washing it with TBST three times, followed by re-blocking the membrane for 1 h.

**Table 1:** List of antibodies.

Antibody	Company	Lot number	Dilute	Source
P-P38 (Thr180/Tyr182)	Cell signaling technology	4,511	1/1,00	Rabbit
P38	Cell signaling technology	9,212	1/1,000	Rabbit
P-PI3K (Tyr458)	Cell signaling technology	4,228	1/1,000	Rabbit
PI3K	Cell signaling technology	4,257	1/1,000	Rabbit
P-AKT (Ser473)	Cell signaling technology	4,060	1/1,000	Rabbit
AKT	Cell signaling technology	4,691	1/1,000	Rabbit
P-GSK3β(Ser9)	Cell signaling technology	5,558	1/1,000	Rabbit
GSK3β	Cell signaling technology	12,456	1/1,000	Rabbit
P-AMPKα(Thr172)	Cell signaling technology	2,535	1/1,000	Rabbit
AMPKα	Cell signaling technology	5,832	1/1,000	Rabbit
P-EEF2 (Thr56)	Cell signaling technology	2,331	1/1,000	Rabbit
EEF2	Abcam	Ab33523	1/1,000	Rabbit
Iba1	Cell signaling technology	17,198	1/1,000	Rabbit
GFAP	Cell signaling technology	3,670	1/1,000	Mouse
NLRP3	Cell signaling technology	15,101	1/1,000	Rabbit
Nrf2	Cell signaling technology	12,721	1/1,000	Rabbit
HO-1	Cell signaling technology	70,081	1/1,000	Rabbit
SOD2	Cell signaling technology	13,194	1/1,000	Rabbit
PSD95	Abcam	Ab18258	1/1,000	Rabbit
SNAP25	Cell signaling technology	5,308	1/1,000	Rabbit
SYNAPSIN1	Abcam	Ab254349	1/1,000	Rabbit
GAPDH	Cell signaling technology	5,174	1/1,000	Rabbit
β-Actin	Santa Cruz biotechnology	Sc-47,778	1/500	Mouse
HIF-1α	Cell signaling technology	14,179	1/1,000	Rabbit
PHD1	ABclonal	A3730	1/1,000	Rabbit
PHD2	ABclonal	A14557	1/1,000	Rabbit
PHD3	ABclonal	A0851	1/1,000	Rabbit

### Statistical analysis

All the statistical analyses were performed using the GraphPad Prism 8 software. Data are presented as mean ± SEM. One-way analysis of variance (ANOVA) followed by *post hoc* Tukey/Bonferroni Multiple Comparison tests was performed to compare different groups. *p* < 0.05 was regarded as statistically significant.

## Results

### Roxadustat (FG-4592) reversed lipopolysaccharides-induced depressive symptoms

Lipopolysaccharides is a well-known inflammatory agent that can induce depressive-like behaviors in mice (Ali et al., 2020a). Similarly, our LPS-treated mice displayed depressive symptoms, as demonstrated by increased immobility during TST and FST, while decreased sucrose preference for 1% sucrose solution over normal water. However, Roxadustat (FG-4592) treatment significantly attenuated these LPS-induced depressive-like behaviors (Figures 1A,B).

### PI3K Signaling mediated the effects of Roxadustat

**Figure 2:**
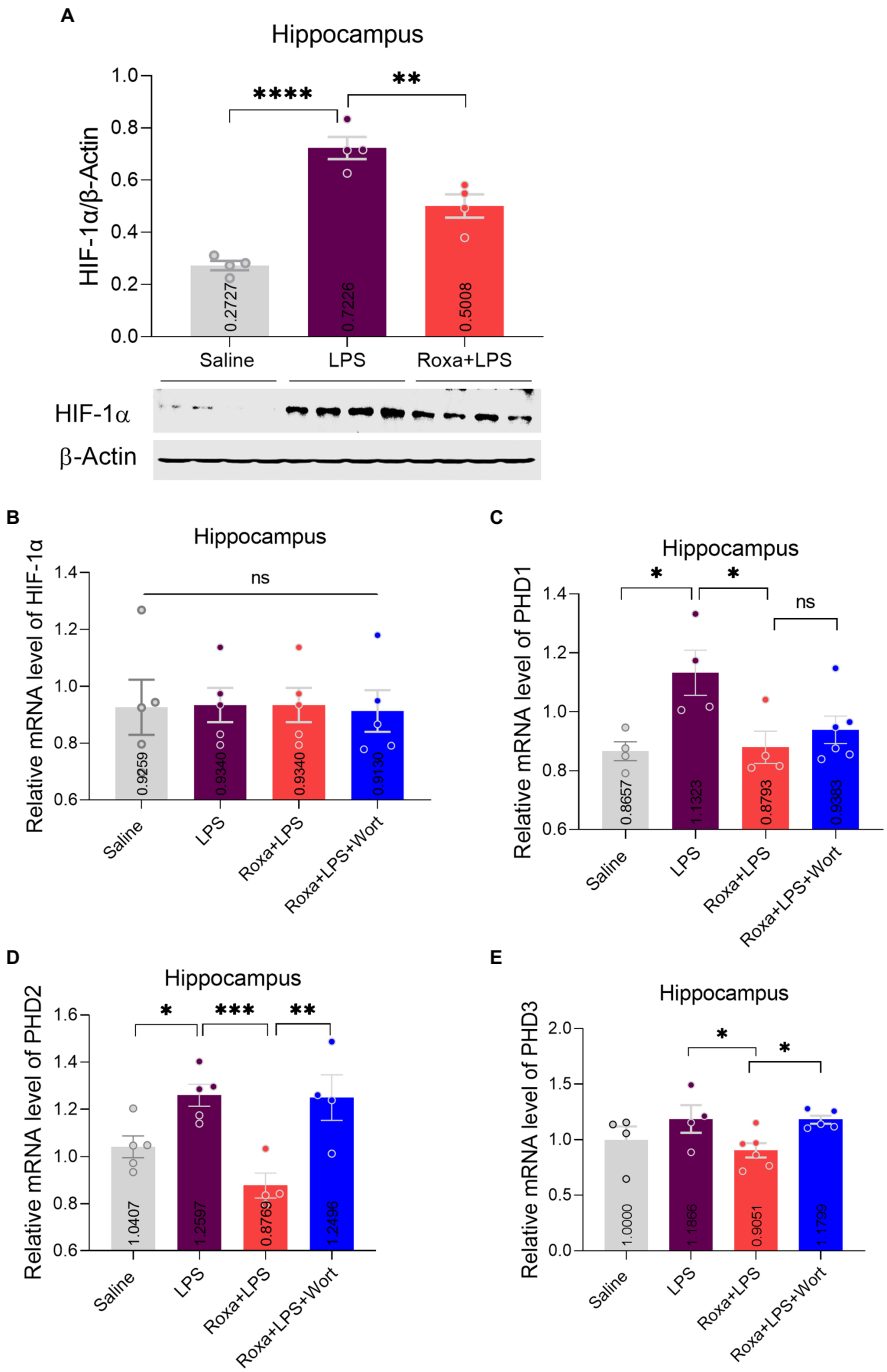
Roxadustat effects on HIF-1a and PHDs expression. **(A)** Representative immune blot images and average protein levels of HIF-1 levels were normalized with β-Actin. **(B–E)** Showing mRNA levels of HIF-1, PHD1, PHD2, and PHD3. Image lab software was used for quantitative blot analysis and was analyzed *via* GraphPad prism. Data were expressed as mean ± SEM, One-way ANOVA, followed by *post hoc* analysis. *p* = <0.05 were considered significant. *p < 0.05, **p < 0.01, ***p < 0.001, ****p < 0.0001. ns: non-significant.

Previous studies evidenced LPS-altered PI3K signaling (Saponaro et al., 2012; Zheng et al., 2018). However, its association with HIF-1 is enigmatic. Herein, we treated mice with wortmannin to determine the interplay among LPS, HIF-1, and PI3K signaling in depression. Surprisingly, PI3K antagonism significantly reversed Roxadustat anti-depressive effects in the presence of LPS, as it found that increased mice immobility decreased sucrose preference (Figures 1C,D). These findings suggested the involvement of HIF-1 and PI3K signaling in LPS-induced depressive symptoms. Moreover, we found an increase in HIF-1 expression, PHD1,2 (mRNA), and PI3K phosphorylation upon LPS administration, which was reduced by Roxadustat treatment (Figure 2; Supplementary Figures S1B,C). As the PI3K-Akt signaling could be activated by LPS stimulus (Hemmings and Restuccia, 2012), we examined Akt and its downstream signaling changes in the hippocampus of LPS-treated mice. Notably, LPS administration significantly enhanced Akt/GSK3β phosphorylation which was reduced by Roxadustat treatment.

**Figure 3:**
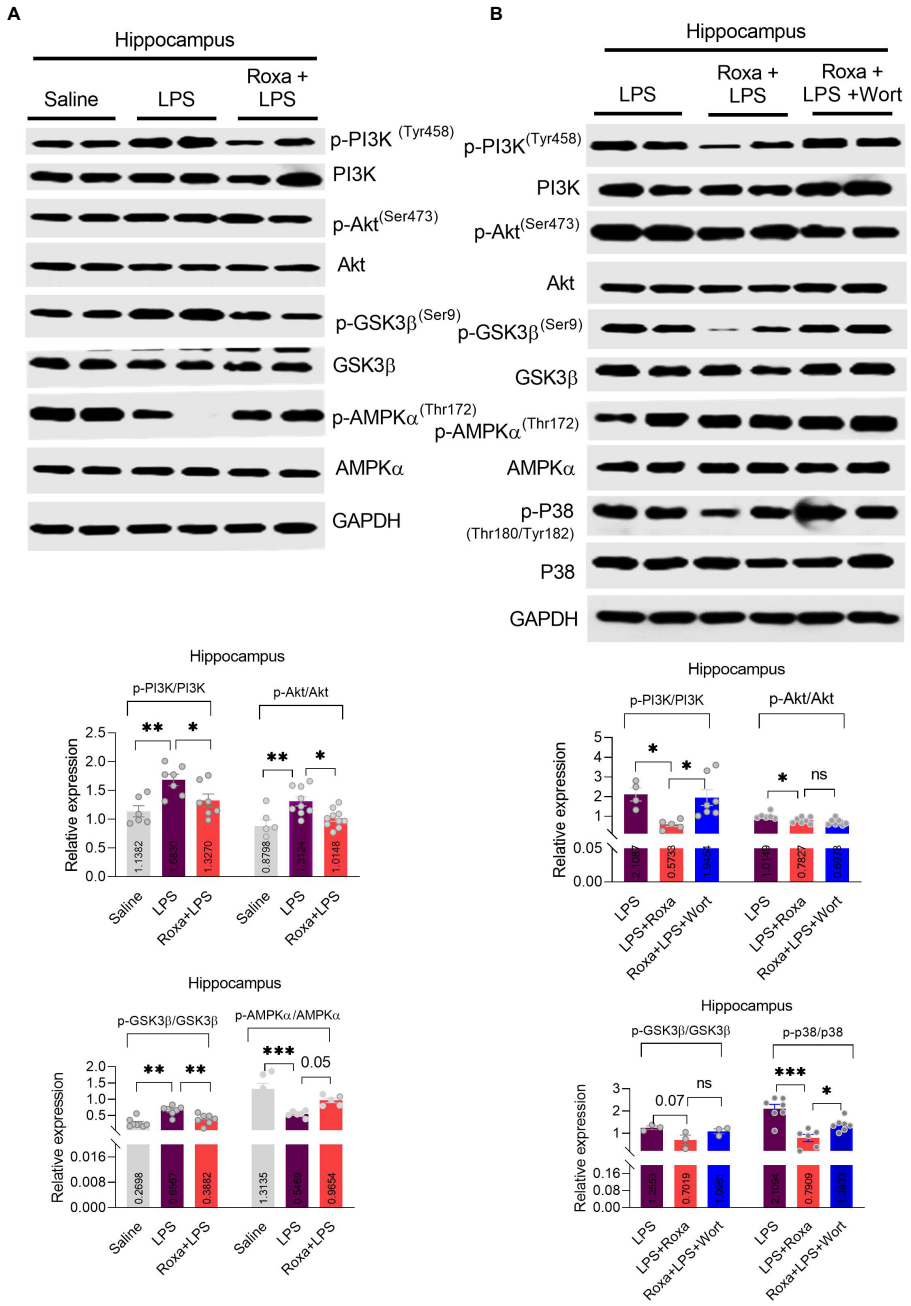
Roxadustat reduced LPS-induced PI3K and its associated signaling. (**A,B)** representative western blots of PI3K, Akt, GSK3β, APMKα, and P38 and quantitative column graphs for mice treated with LPS, Roxadustat and wortmannin. All the values were normalized with GAPDH. Image lab software was used for blots quantitative analysis and was analyzed *via* GraphPad prism. Data were expressed as mean ± SEM, One-way ANOVA, followed by *post hoc* analysis. *p* = <0.05 were considered significant. *p < 0.05, **p < 0.01), ***p < 0.001. ns: non-significant.

Contrarily, Roxadustat treatment significantly increased AMPKα phosphorylation in the presence of LPS (Figure 3A), indicating a link between HIF-1 and AMPK singling under LPS-induced stress conditions. As reported, AMPK is involved in the Akt signaling activation (Han et al., 2018); its link to PI3K is largely unknown, particularly in LPS-initiated stress conditions. Thus, we examined AMPKα/Akt/GSK3b signaling changes upon PI3K antagonism in the presence of LPS. Except for Akt, wortmannin treatment significantly attenuated Roxadustat-induced p-AMPKα, GSK3b, and p-p38 expressional changes in the mice hippocampal tissues in the presence of LPS (Figure 3B). These findings indicated the interplay among AMPK/PI3K and HIF-1 signaling under LPS-induced stress conditions.

### Roxadustat treatment attenuated lipopolysaccharides-induced neuroinflammation

**Figure 4:**
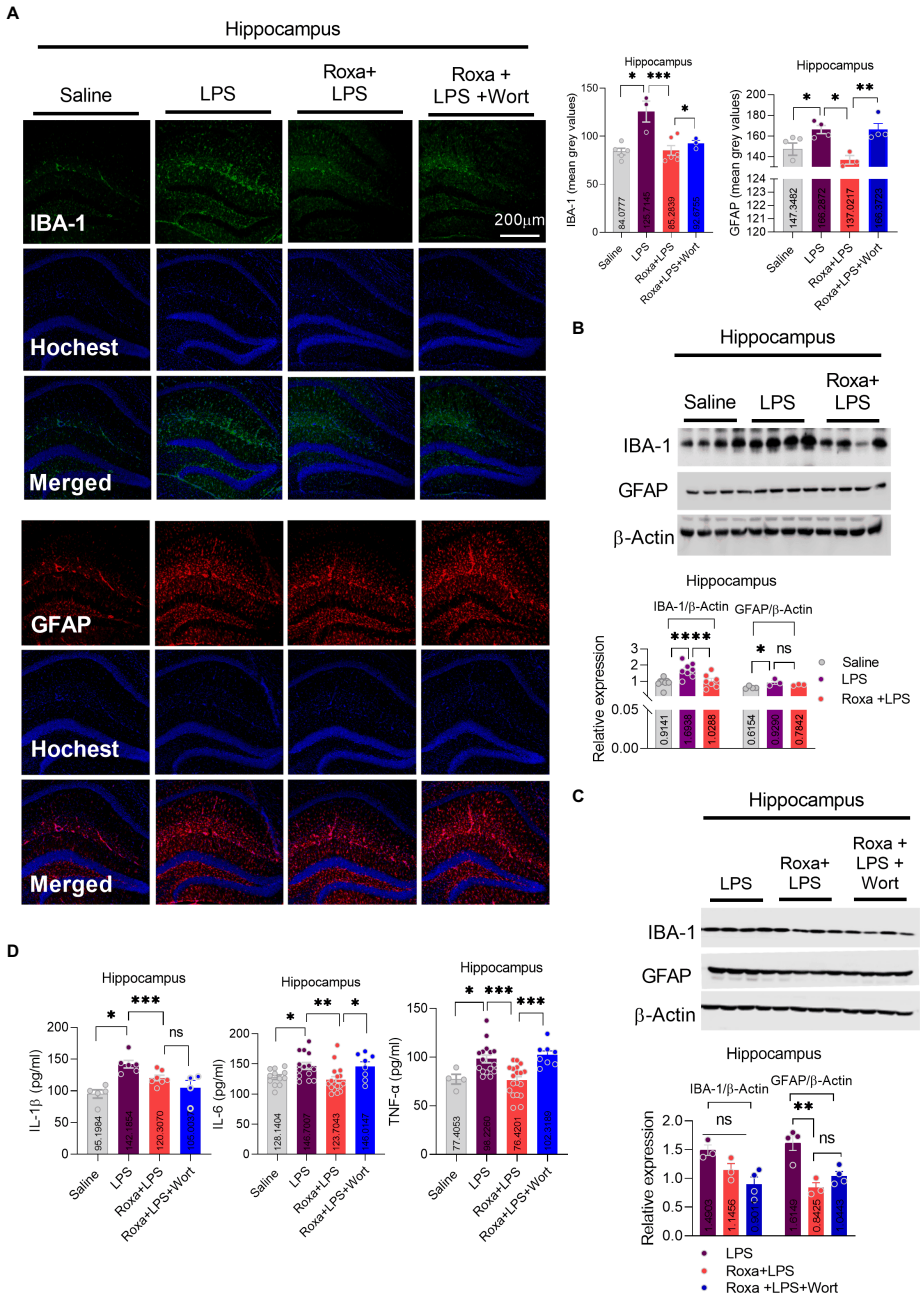
Roxadustat reduced LPS-induced neuroinflammation. **(A)** Microscopy results of Iba-1 expression in the different experimental groups of brain tissues, with respective bar graphs (*n* = 6). Magnification 10×. The image data were collected from three independent experiments and were analyzed by ImageJ software. The differences are shown in the graphs. **(B,C)** Representative immune blots with individual level column graphs showing Iba-1 and GFAP expression *n* = 3–6. All the values were normalized with β-Actin. **(D)** Bar graphs showing the expression level of IL-1β, IL-6, and TNF-α. Data were expressed as mean ± SEM, One-way ANOVA, followed by *post hoc* analysis. *p* = <0.05 were considered significant. *p < 0.05, **p < 0.01, ***p < 0.001, ****p < 0.0001. ns: non-significant.

**Figure 5:**
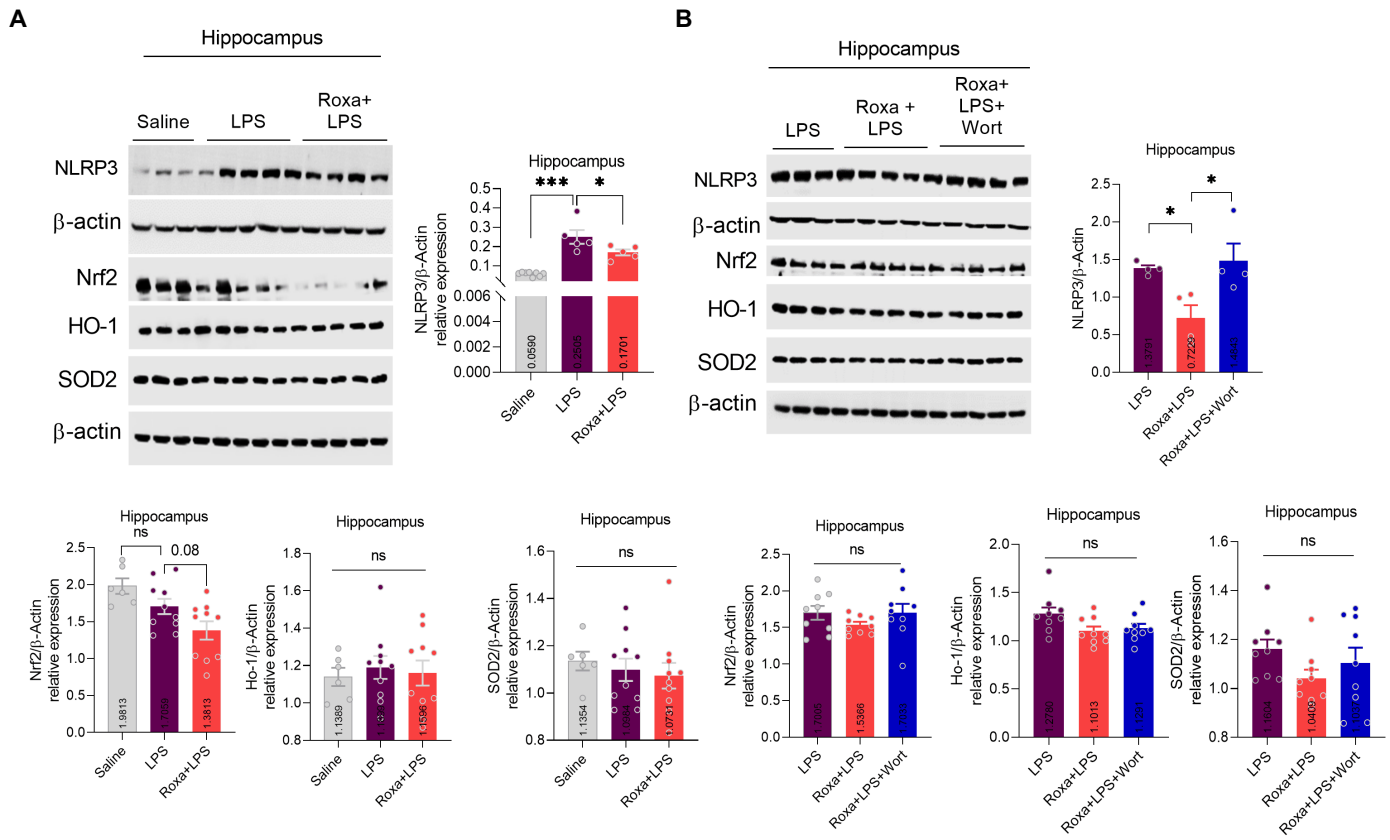
Roxadustat reduced NLRP3 level in the presence of LPS. **(A,B)** Representative immune blots images and bar graphs show the expression of NLRP3, Nrf2, HO-1, and SOD2. All the values were normalized with β-Actin. Image lab software was used for blots quantitative analysis and was analyzed *via* GraphPad prism. Data were expressed as mean ± SEM, One-way ANOVA, followed by *post hoc* analysis. *p* = <0.05 were considered significant, *p < 0.05, ***p < 0.001. ns: non-significant.

Lipopolysaccharides-induced neuroinflammation played an integral role of depressive symptoms in mice (Li et al., 2017, 2021a). Here, we sought to determine whether LPS-induced neuroinflammation is linked to HIF-1 signaling. Our results indicated that Roxadustat significantly reduced IBA-1 and GFAP expression in the hippocampus (DG region) of the LPS-treated mice (Figures 4A,B). Similarly, Roxadustat treatment decreased cytokines, including IL-1b, IL-6, and TNF-a levels in the hippocampal tissue of the brain (Figure 4D). However, the anti-inflammatory effects of Roxadustat could be attenuated by wortmannin treatment (Figures 4A,C,D), suggesting PI3K signaling was involved in HIF-1 modulation of LPS-induced neuroinflammation. The cellular signaling changes associated with neuroinflammation were examined to validate these results further. Surprisingly, Roxadustat treatment significantly reduced LPS-elevated expression of NLRP3 in mice brains (Figure 5A), which could be increased by wortmannin (Figure 5B). As LPS-induced inflammation accompanies oxidative stress, we thus measured the expressions of anti-oxidative markers, including Nrf2, SOD2, and HO-1. However, no significant changes in NRF2, SOD2, and HO-1 Roxadustat and Wortmannin treatment could be defined (Figures 5A,B).

### HIF-1-PHD antagonism by Roxadustat attenuated lipopolysaccharides-induced synaptic defects

**Figure 6:**
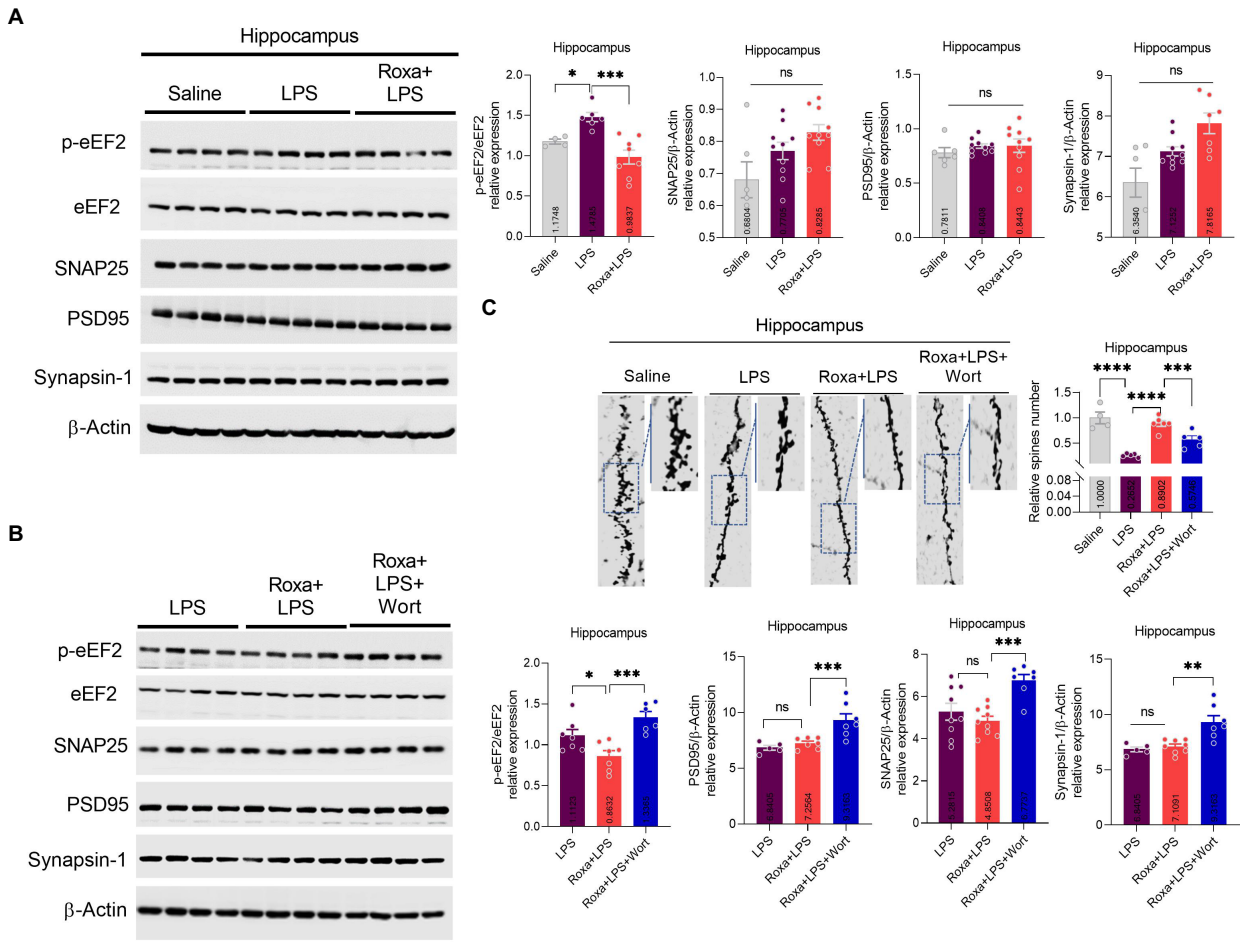
Changes in the expression of eEF2, SNAP25, PSD95, and Synapsin-1, and spine numbers after Roxa and LSP treatment. **(A,B)** Representative immune blots show the expression of p-eEF2, eEF2, SNAP25, PSD95, and Synapsin-1 levels in the hippocampus of subjects. All the values were normalized with β-Actin. (**C)** Golgi staining showing spine density and column graph showing spine numbers *n* = 3–5. Image lab software was used for blots quantitative analysis and was analyzed *via* GraphPad prism. Data were expressed as mean ± SEM, One-way ANOVA, followed by *post hoc* analysis. *p* = <0.05 were considered significant. *p < 0.05, **p < 0.01), ***p < 0.001, ****p < 0.0001. ns: non-significant.

Previous studies, including ours, showed that LPS administration could induce synaptic defects, which may underly the pathological processes of depression symptoms (Postnikova et al., 2020; Li et al., 2021a). Our results revealed dysregulated synaptic factors and reduced spins in hippocampal tissues (Figure 6C). Besides, the eEF2 phosphorylation level, which regulates synaptic plasticity, was also increased in LPS-treated mice but reversed by Roxadustat treatment (Figure 6A; Verpelli et al., 2010). Moreover, wortmannin attenuated the effects of Roxadustat and enhanced synaptic proteins, including PSD95, SNAP25, and Synapsin-1 (Figure 6B). These findings suggest that Roxadustat could alleviate dysregulated synaptic proteins induced by LPS *via* HIF-1/PI3K signaling.

## Discussion

Results from our previous studies and other groups have documented the interplay between neuroinflammation and depressive-like behaviors (Li et al., 2017; Postnikova et al., 2020; Ali et al., 2020b; Li et al., 2021b), but its mechanistic link to HIF-1/PI3K signaling remains poorly explored. Here, we determined that LPS administration dysregulated HIF-1 *via* the PI3K pathway in the mice hippocampus, which underlined LPS elicited neuroinflammation and depressive-like behaviors. HIF-1-PHD inhibitor (Roxadustat) treatment attenuated LPS-mediated changes, which could be reversed by wortmannin, suggesting an etiological role of HIF-1 in neuroinflammation-associated depressive-like behaviors. However, conventional antidepressants show their effects slowly and may take about 2–3 weeks (Szegedi et al., 2009), immediate/quick antidepressant effects of several compounds/drugs (including Roxadustat treated here for 3 days) have also been documented in the LPS-induced model of depression (O’Connor et al., 2009; Cordeiro et al., 2019; Zhang et al., 2019).

As a transcriptional factor, HIF-1 controls the immune cell metabolism and function. Additionally, it plays a crucial role in regulating inflammatory functions in dendritic, mast, and epithelial cells (Imtiyaz and Simon, 2010). Besides, TNF-α and IL-1β can activate and increase HIF-1 expression/transcriptional *via* NF-kB (Jung et al., 2003; Zhou et al., 2003), indicating that HIF-1 could play an essential role in inflammation. Furthermore, it has also been reported that LPS can stimulate HIF-1 activities *via* several pathways, including NF-kB, ROS, and p42/p44 mitogen-activated protein kinases (MAPKs; Frede et al., 2006). In addition, in macrophages, LPS accumulates HIF-1 *via* decreasing PHD2 and PHD3 levels in a Toll-like receptor-4 (TLR-4) dependent manner (Peyssonnaux et al., 2007; Imtiyaz and Simon, 2010). This evidence supports our findings that LPS-induced neuroinflammation was reduced in the hippocampus of mice after Roxadustat treatment. However, it was more interesting to be identified here that PI3K inhibition reversed the effects of Roxadustat, indicating a link between HIF-1 and PI3K signaling in the presence of LPS. Previous studies have also demonstrated that PI3K/Akt signaling could regulate HIF-1 levels *via* mTOR signaling at the posttranslational level but not at the mRNA level (under hypoxic conditions; Zhang et al., 2018). Because PI3K inhibitor (LY294002) and Dual PI3K/mTOR inhibitor NVP-BEZ235 treatment suppressed Akt and HIF-1 activation and expression (Karar et al., 2012), respectively. These results indicated that the PI3K-Akt cascade is a highly conserved intracellular signaling pathway involved in the immune system’s growth, motility, survival, metabolism, and coordinating defense mechanisms by transducing extracellular stimuli (Hemmings and Restuccia, 2012).

Studies demonstrate the beneficial effects of intermittent hypoxia on neurological disorders, including depression, by promoting neurogenesis *via* BDNF signaling (Ferrari et al., 2017; Meng et al., 2020); however, the mechanisms are not fully explored as for the potential beneficial effects through the HIF-1 hypoxia responding factor. Our results showed that Roxadustat treatment rescued LPS-altered synaptic protein level and spin-altered morphology. Interestingly, similar neuroprotective roles of Roxadustat have been recently reported, while its link to LPS/PI3K signaling has not been explored. Our results further demonstrated that PI3K signalling-antagonism reversed the protective effects of Roxadustat, indicating that HIF-1/PI3K signaling mediated the LPS-induced neuroinflammation, synaptic deficits, and depressive symptoms.

In conclusion, HIF-1 contributed to inflammation and the redox system under hypoxia conditions; it also played a role in neuroinflammation-induced depression, which might be associated with PI3K-related signalings. Furthermore, Roxadustat showed potent anti-depressive effects *via* reducing neuroinflammation by stabilizing HIF-1 expression; however, the proper LPS-stimulated signaling cross-talk to HIF-1 under depression conditions is enigmatic, which is the limitation of the present study, and it needs further investigation.

## Data availability statement

The original contributions presented in the study are included in the article/Supplementary material, further inquiries can be directed to the corresponding authors.

## Ethics statement

The animal study was reviewed and approved by Institutional Animal Care and Use Committee of Peking University, Shenzhen Graduate School.

## Author contributions

AL, TA, and SL: conceptualization. AL, ZL, RG, YL, QG, and HG: methodology. AL, ZL, CZ, WL, XL, and TL: investigation, analysis and funding support. TA and SL: writing. SL and TL: supervision. All authors reviewed and approved the manuscript.

## Funding

This work was supported by the National Natural Science Foundation of CHINA (NSFC grant numbers: 82072112 and 81871530).

## Conflict of interest

The authors declare that the research was conducted in the absence of any commercial or financial relationships that could be construed as a potential conflict of interest.

## Publisher’s note

All claims expressed in this article are solely those of the authors and do not necessarily represent those of their affiliated organizations, or those of the publisher, the editors and the reviewers. Any product that may be evaluated in this article, or claim that may be made by its manufacturer, is not guaranteed or endorsed by the publisher.
