# Supplementary material for: Roxadustat (FG-4592) abated lipopolysaccharides-induced depressive-like symptoms via PI3K signaling
Source: Front Mol Neurosci. 2023 Mar 15;16:1048985. doi: 10.3389/fnmol.2023.1048985 (PMC10056220; doi:10.3389/fnmol.2023.1048985)

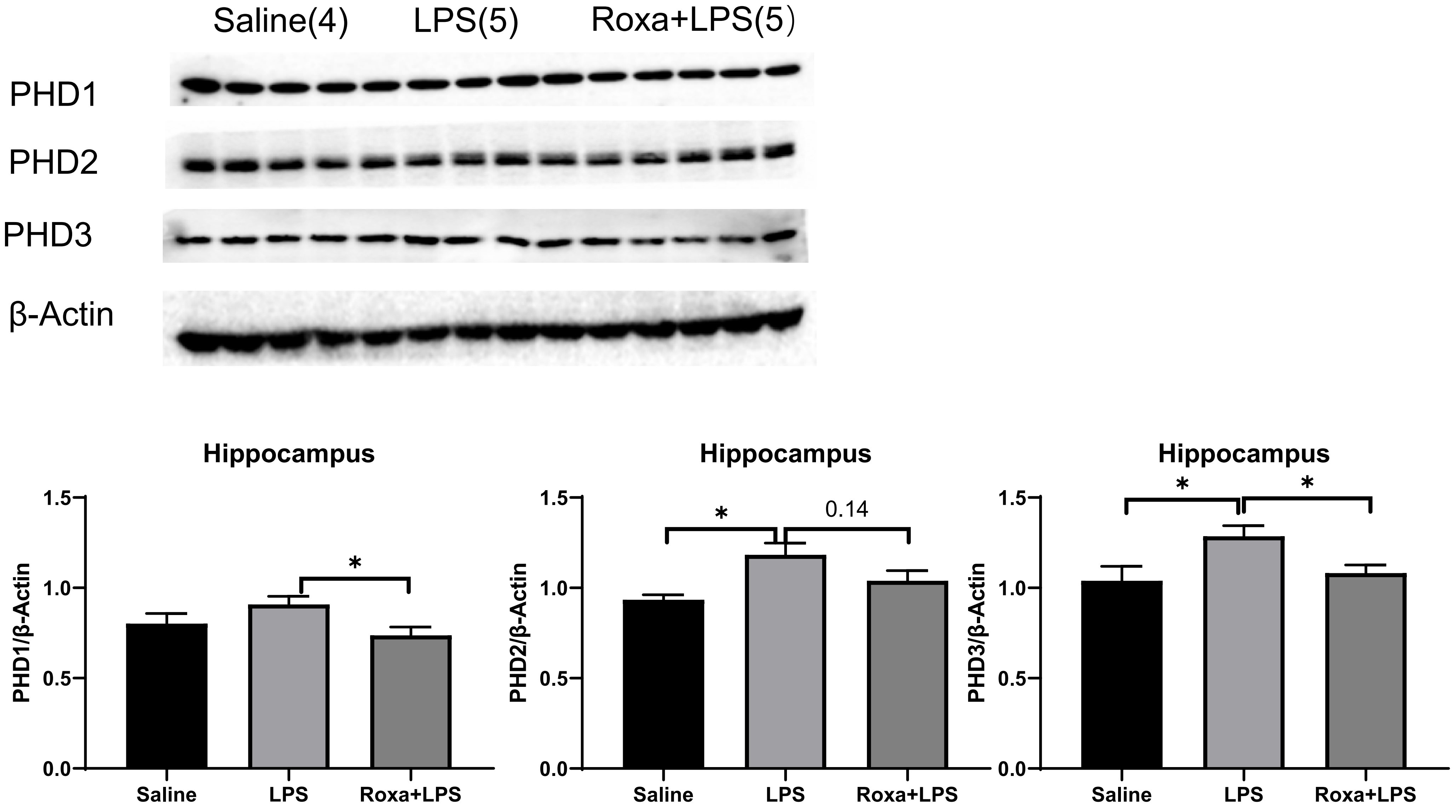

Saline(4)
LPS(5)
Roxa+LPS(5)
PHD1
PHD2
PHD3
β-Actin
Hippocampus
PHD1/β-Actin
1.5
1.0
0.5
0.0
*
Saline
LPS
Roxa+LPS
Hippocampus
PHD2/β-Actin
1.5
1.0
0.5
0.0
*
0.14
Saline
LPS
Roxa+LPS
Hippocampus
PHD3/β-Actin
1.5
1.0
0.5
0.0
*
*
Saline
LPS
Roxa+LPS

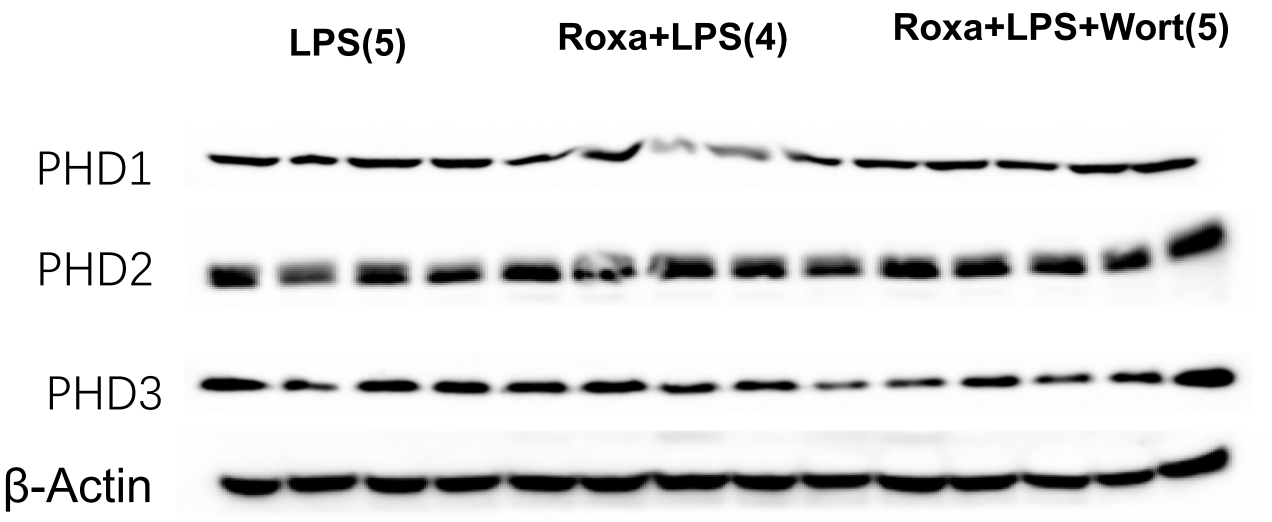

Maybe we can delete PHD2 because this time Roxa+LPS did not decrease PHD2

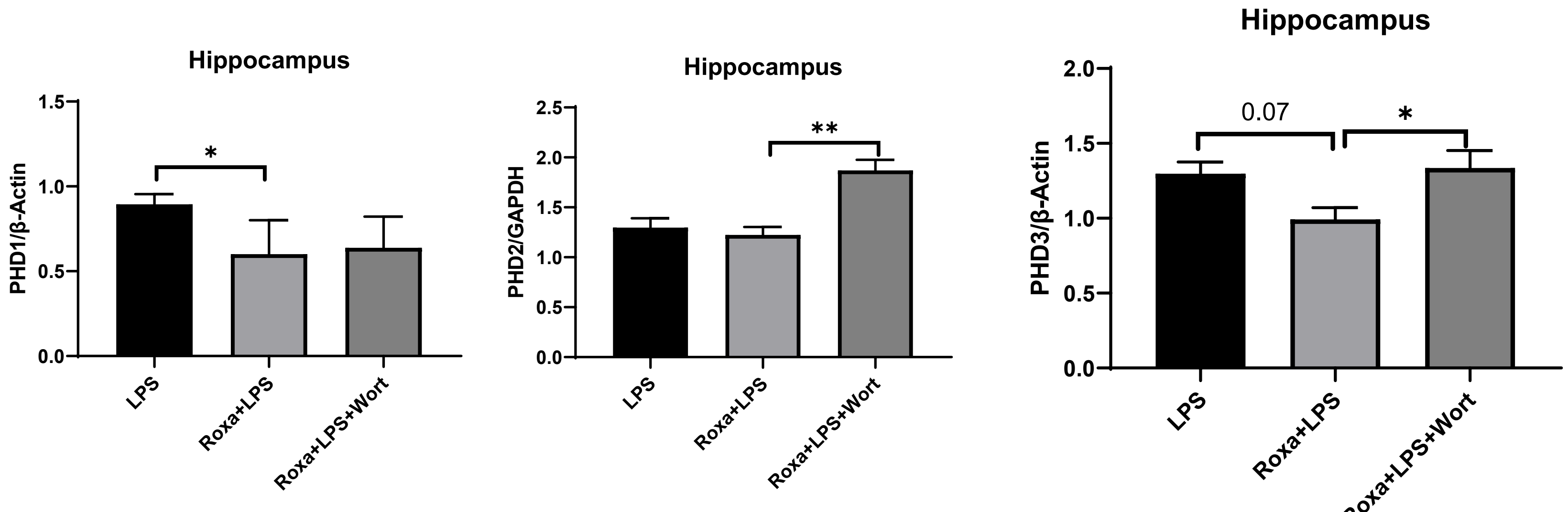

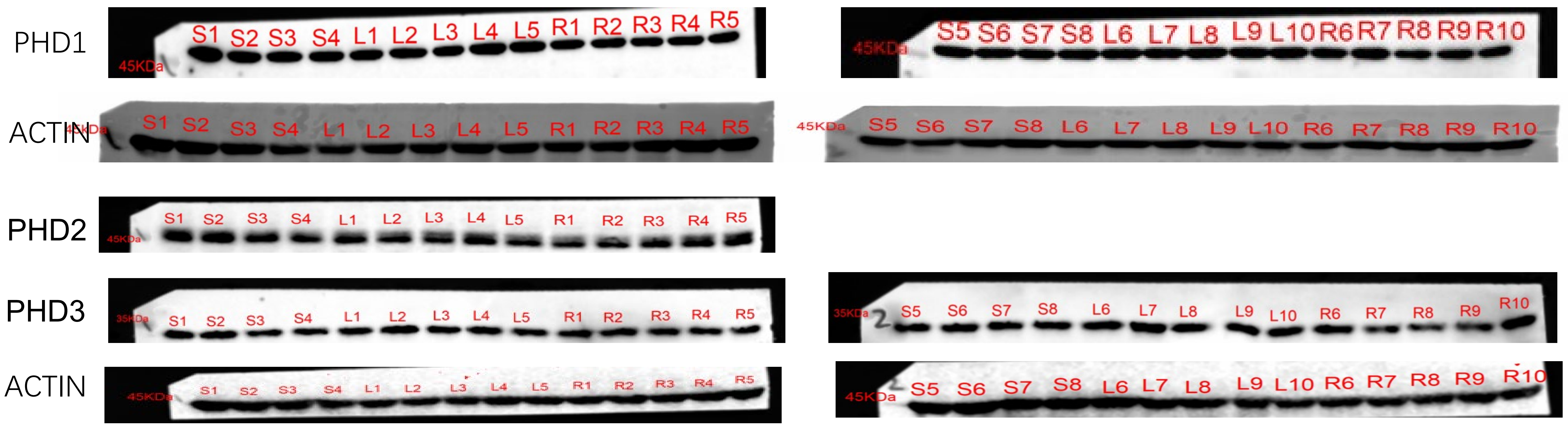
PHD1
45KDa
S1 S2 S3 S4 L1 L2 L3 L4 L5 R1 R2 R3 R4 R5
45KDa
S5 S6 S7 S8 L6 L7 L8 L9 L10 R6 R7 R8 R9 R10
ACTIN
45KDa
S1 S2 S3 S4 L1 L2 L3 L4 L5 R1 R2 R3 R4 R5
45KDa
S5 S6 S7 S8 L6 L7 L8 L9 L10 R6 R7 R8 R9 R10
PHD2
45KDa
S1 S2 S3 S4 L1 L2 L3 L4 L5 R1 R2 R3 R4 R5
PHD3
35KDa
S1 S2 S3 S4 L1 L2 L3 L4 L5 R1 R2 R3 R4 R5
35KDa
S5 S6 S7 S8 L6 L7 L8 L9 L10 R6 R7 R8 R9 R10
ACTIN
45KDa
S1 S2 S3 S4 L1 L2 L3 L4 L5 R1 R2 R3 R4 R5
45KDa
S5 S6 S7 S8 L6 L7 L8 L9 L10 R6 R7 R8 R9 R10

Statistic analyse blots

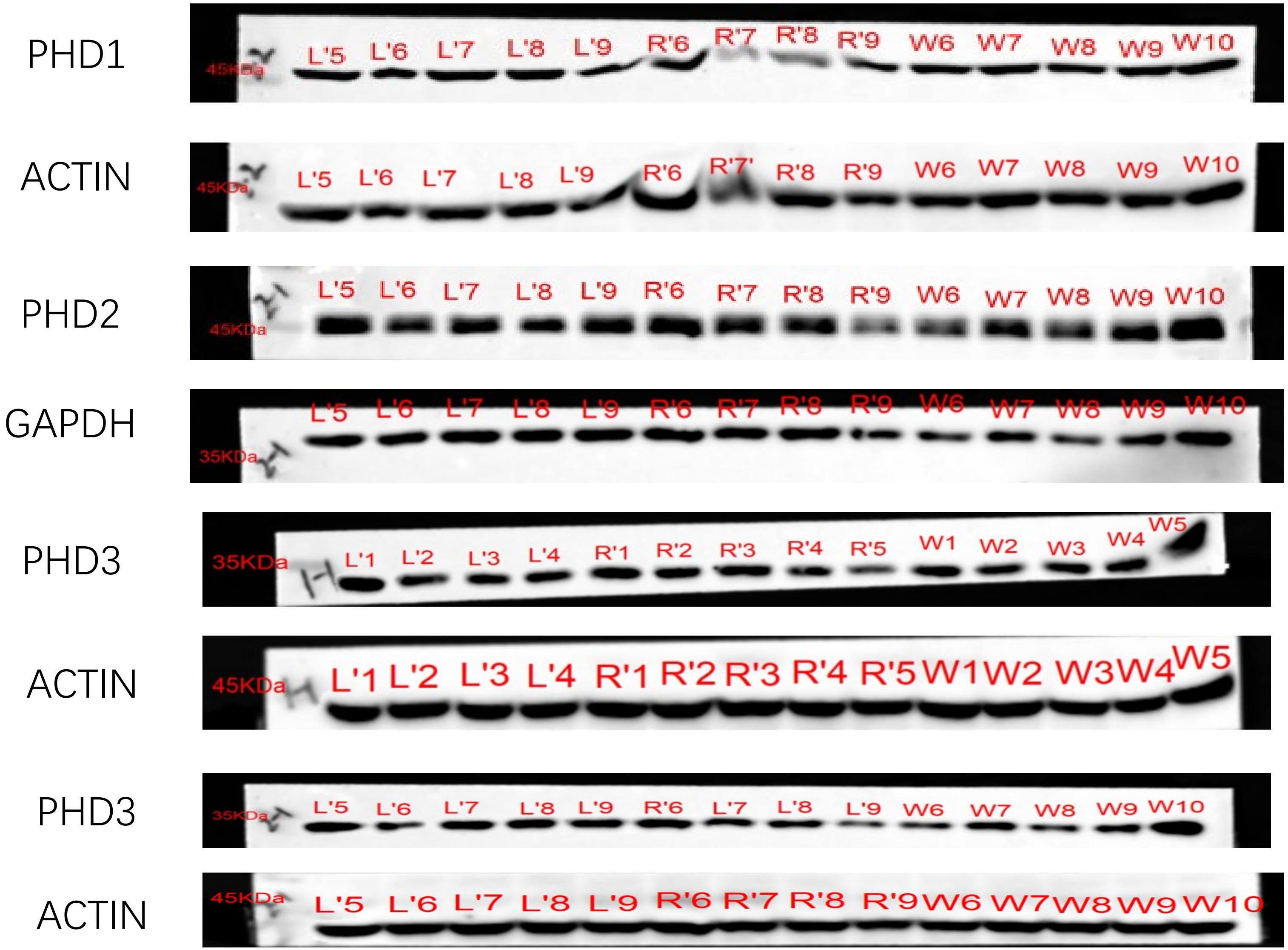

Supplement: Supplementary file 1 [file Data_Sheet_1.PDF]
