# Supplementary material for: Roxadustat (FG-4592) abated lipopolysaccharides-induced depressive-like symptoms via PI3K signaling
Source: Front Mol Neurosci. 2023 Mar 15;16:1048985. doi: 10.3389/fnmol.2023.1048985 (PMC10056220; doi:10.3389/fnmol.2023.1048985)
Supplement: Supplementary file 2 [file Data_Sheet_2.PDF]

Figure2A

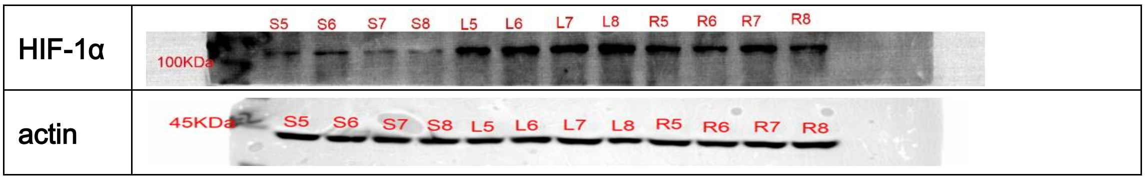

Figure3A

Note: S= saline, L= LPS, R= LPS+Roxa, W= LPS+Roxa+Wort,

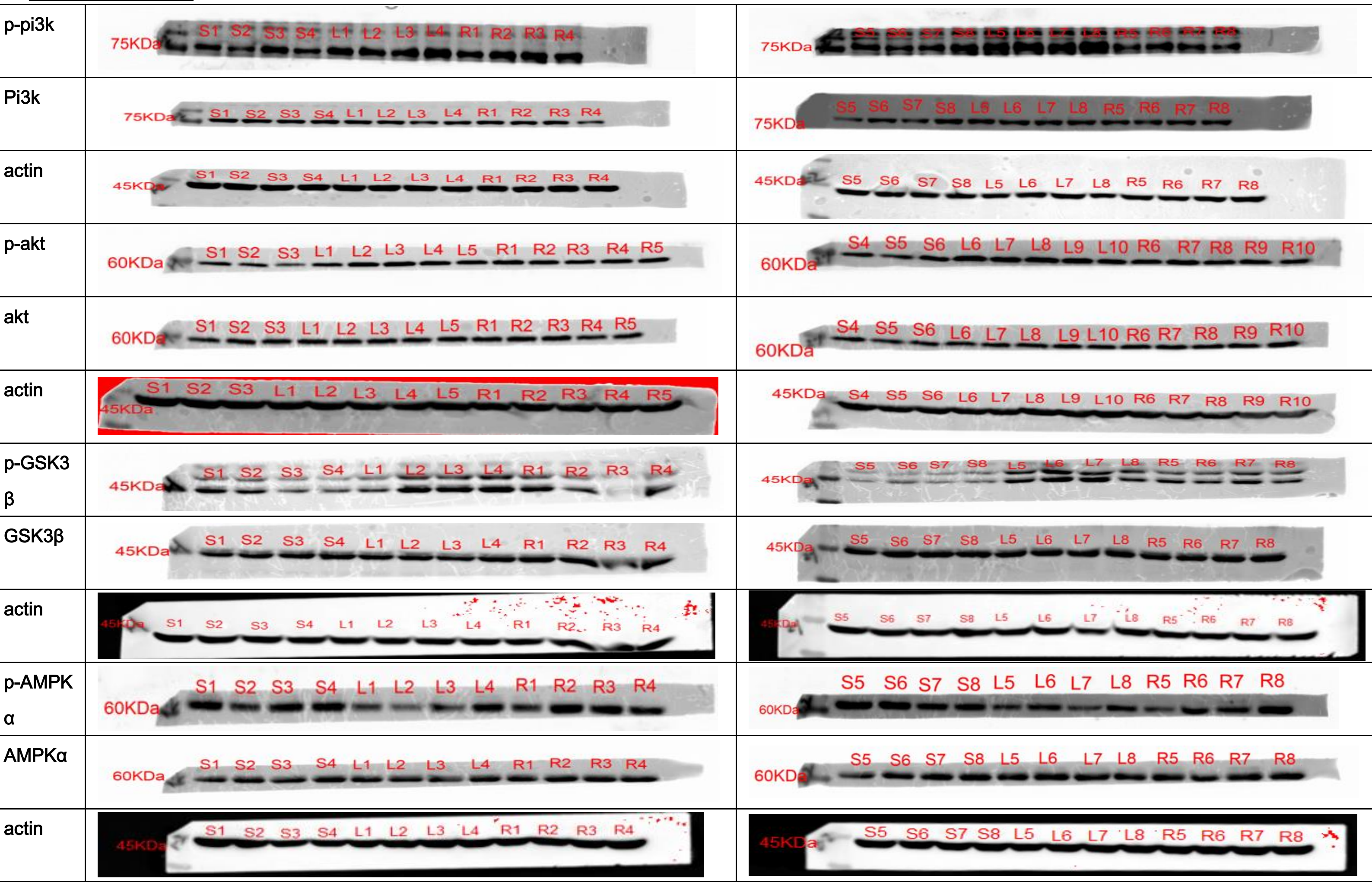

Figure3B

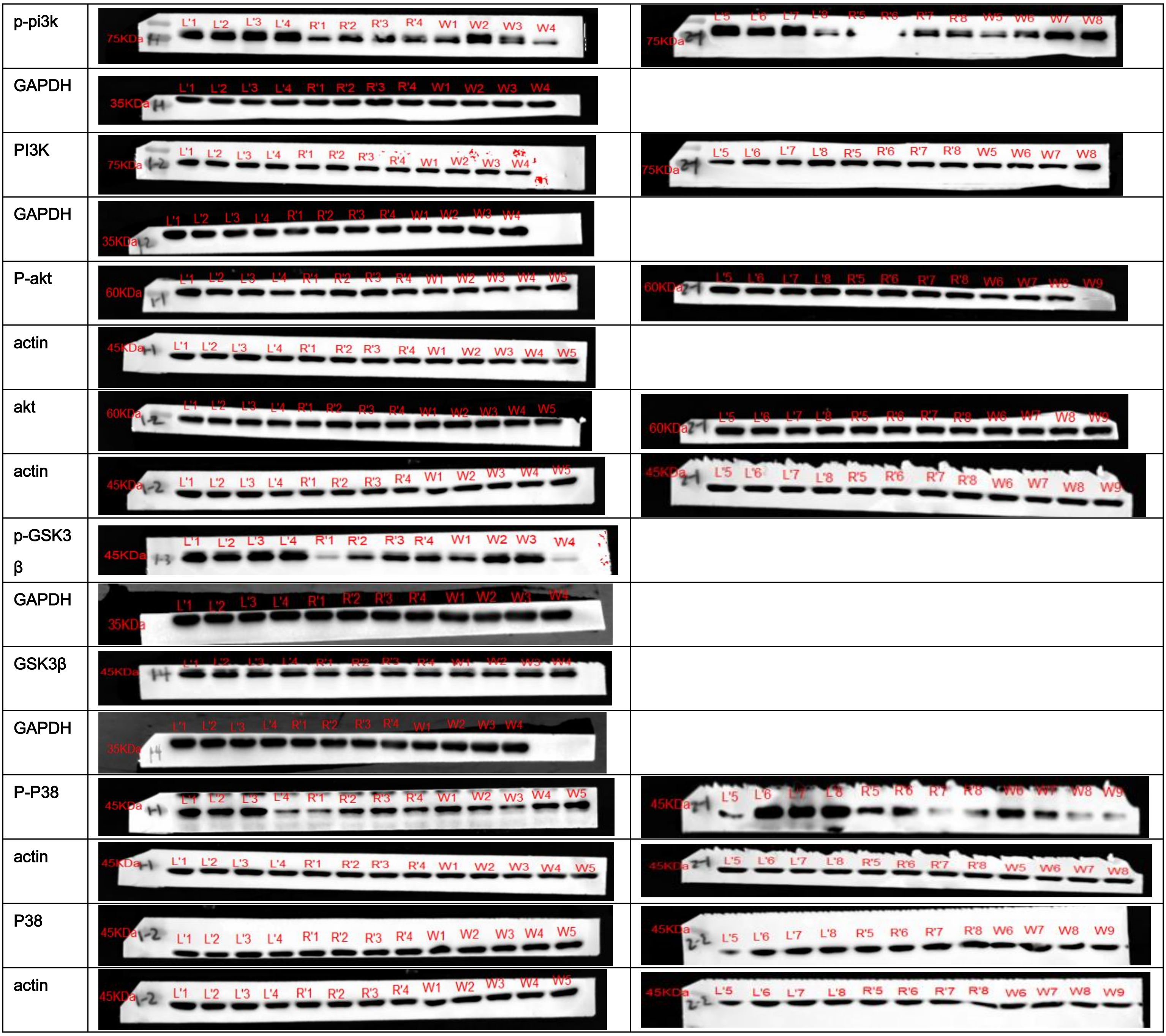
p-pi3k
GAPDH
PI3K
GAPDH
P-akt
actin
akt
actin
p-GSK3β
GAPDH
GSK3β
GAPDH
P-P38
actin
P38
actin

Figure4B

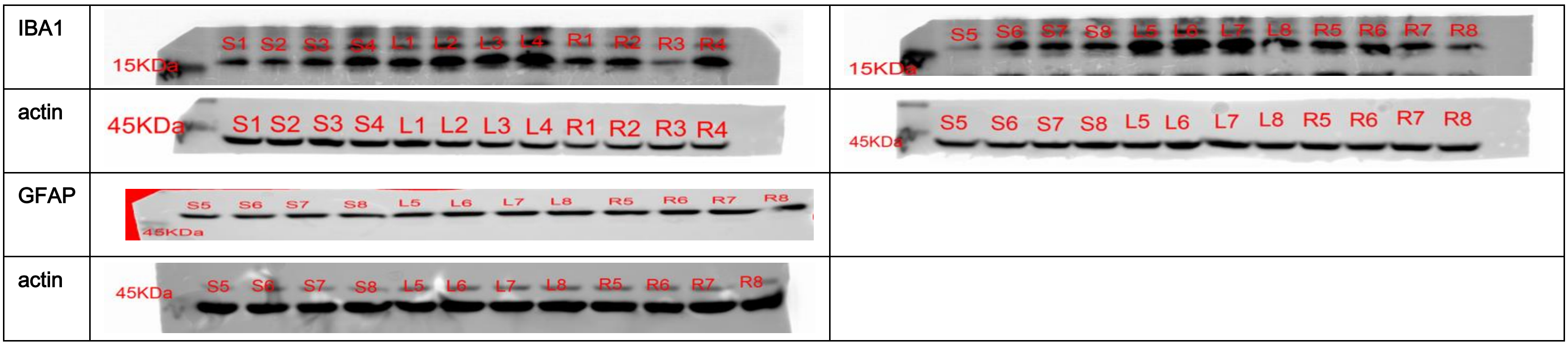

Figure4C

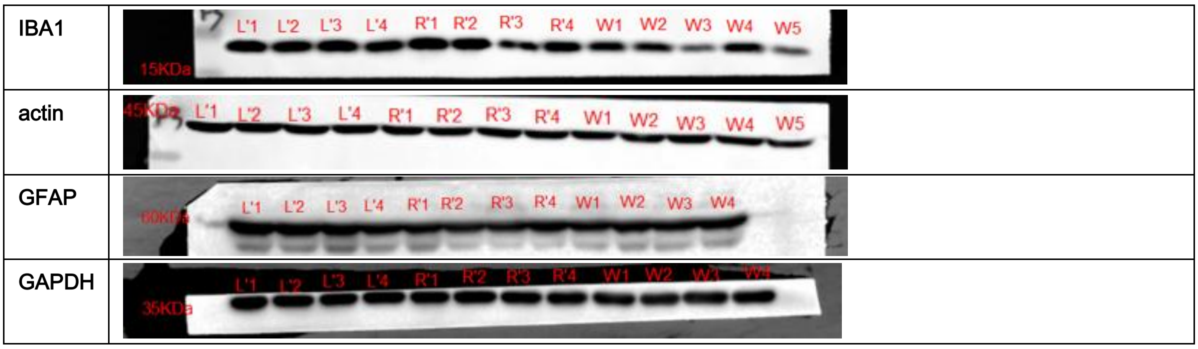

Figure5A

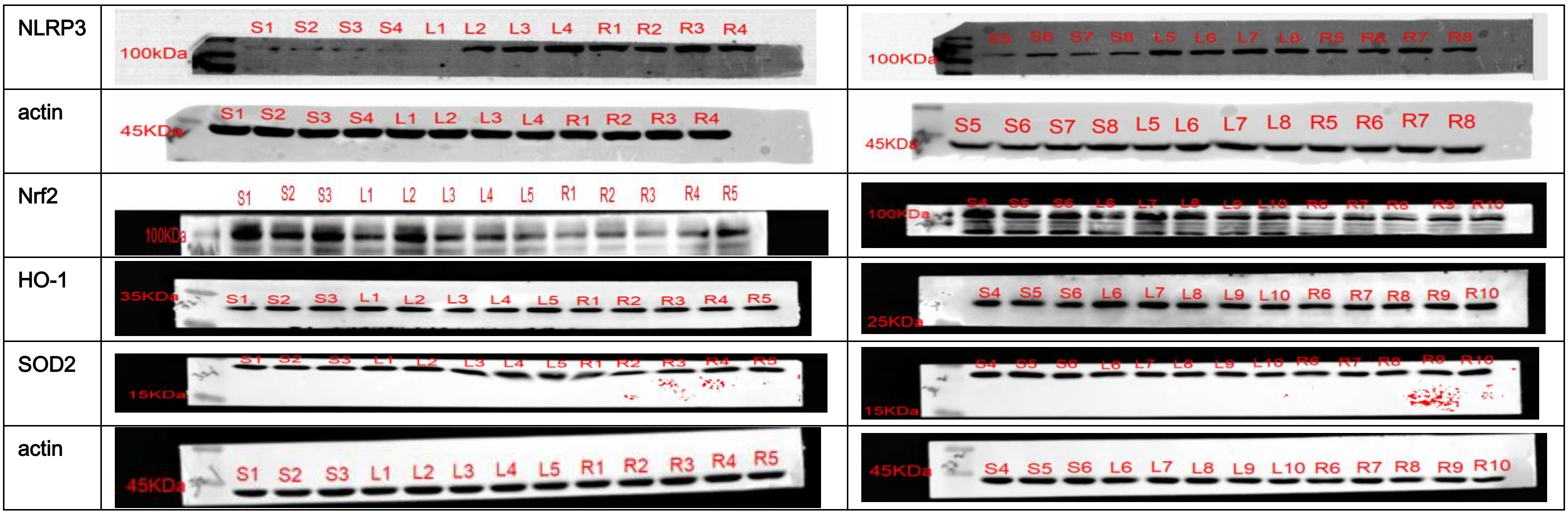

Figure5B

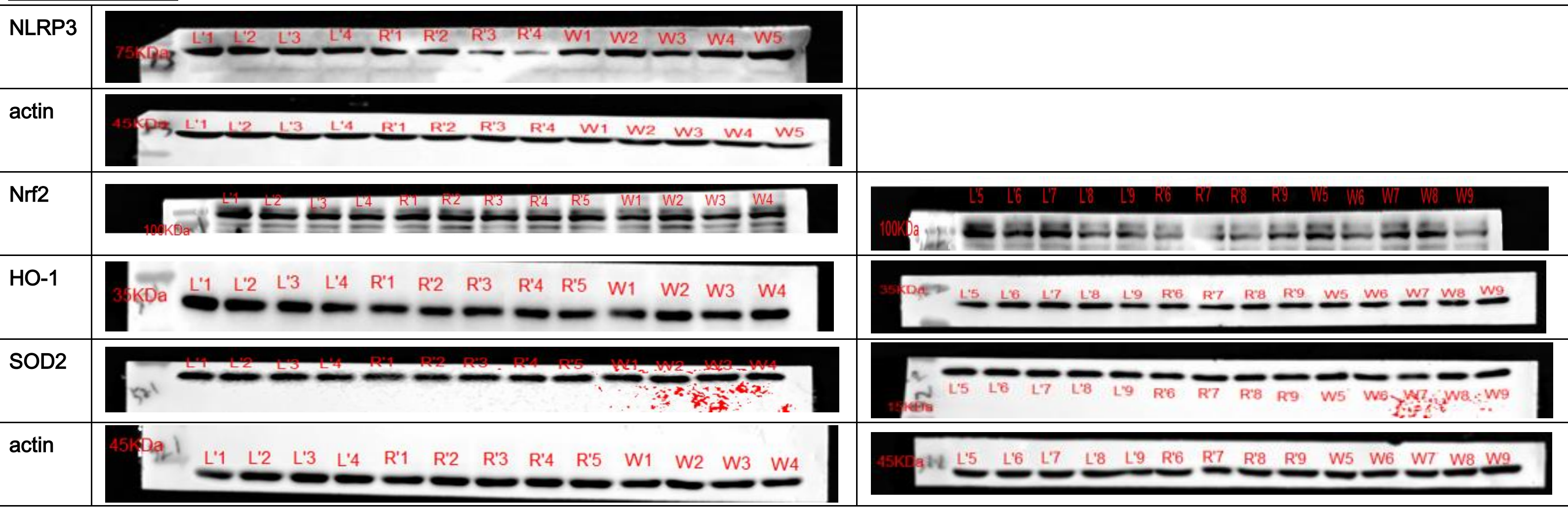

Figure6A

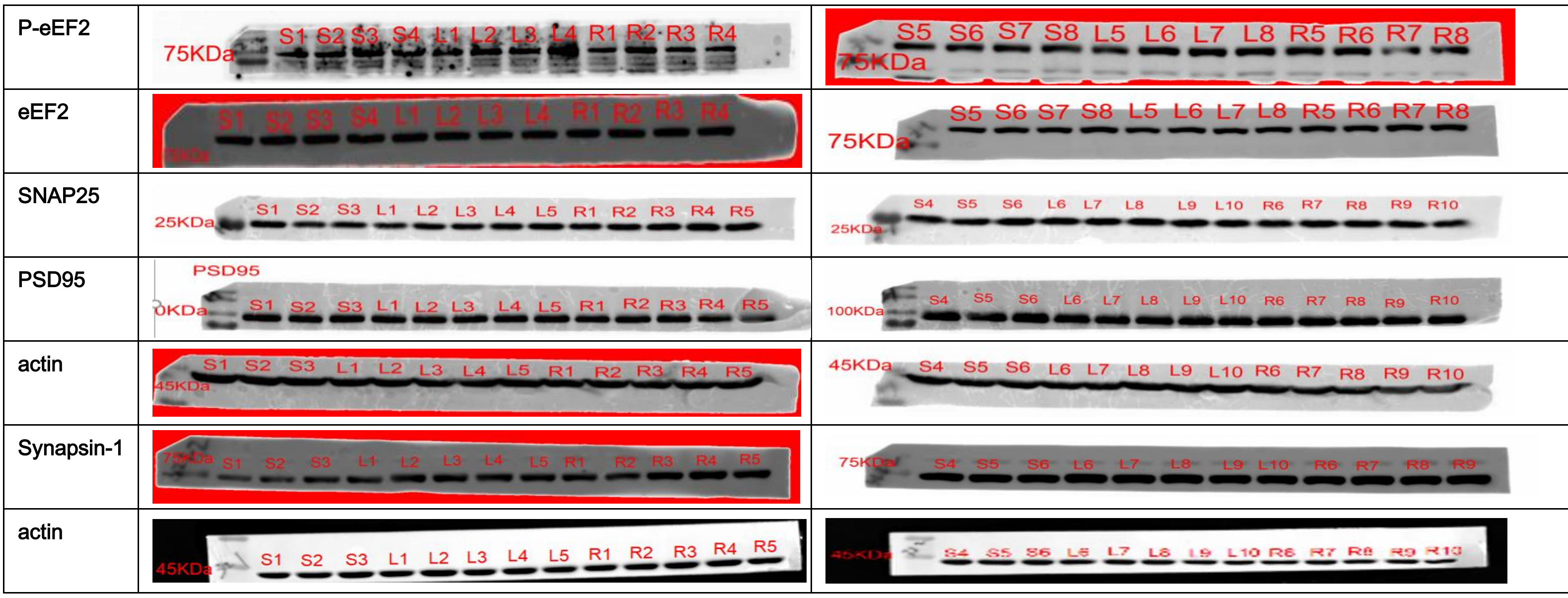

Figure6B

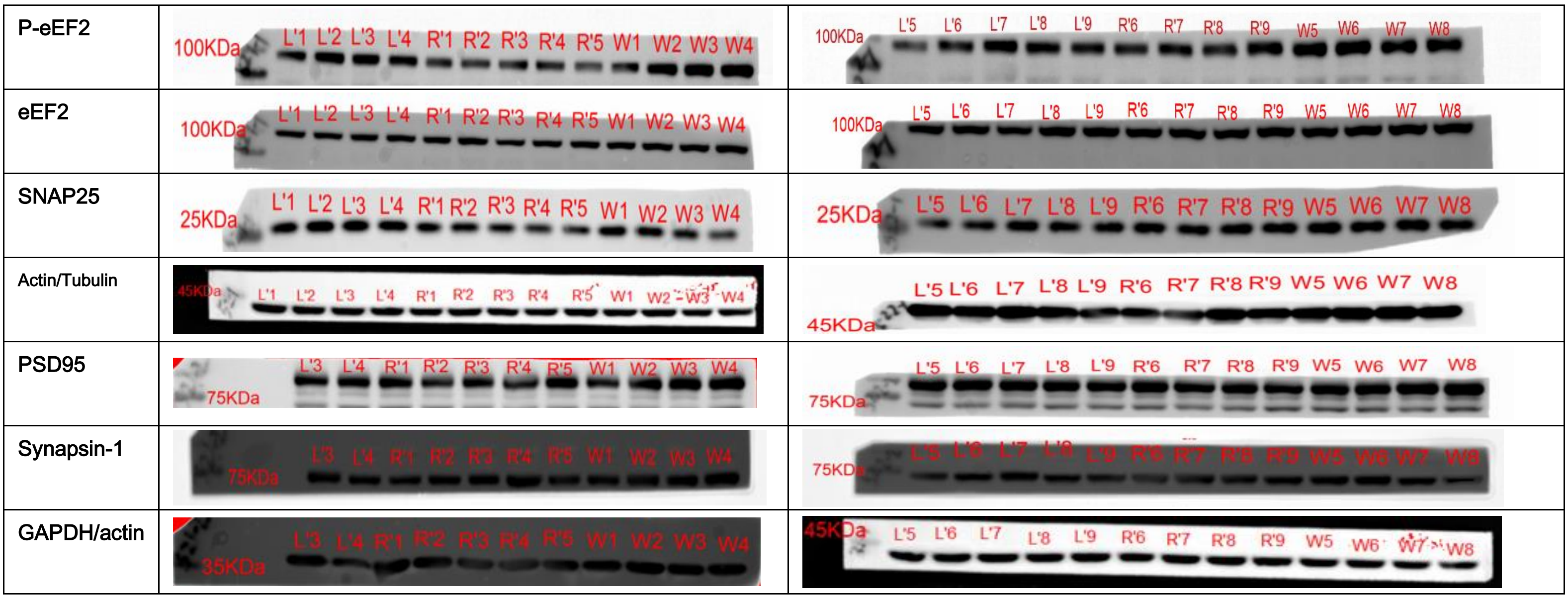
P-eEF2
100KDa L'1 L'2 L'3 L'4 R'1 R'2 R'3 R'4 R'5 W1 W2 W3 W4
100KDa L'5 L'6 L'7 L'8 L'9 R'6 R'7 R'8 R'9 W5 W6 W7 W8
eEF2
100KDa L'1 L'2 L'3 L'4 R'1 R'2 R'3 R'4 R'5 W1 W2 W3 W4
100KDa L'5 L'6 L'7 L'8 L'9 R'6 R'7 R'8 R'9 W5 W6 W7 W8
SNAP25
25KDa L'1 L'2 L'3 L'4 R'1 R'2 R'3 R'4 R'5 W1 W2 W3 W4
25KDa L'5 L'6 L'7 L'8 L'9 R'6 R'7 R'8 R'9 W5 W6 W7 W8
Actin/Tubulin
45KDa L'1 L'2 L'3 L'4 R'1 R'2 R'3 R'4 R'5 W1 W2 W3 W4
L'5 L'6 L'7 L'8 L'9 R'6 R'7 R'8 R'9 W5 W6 W7 W8 45KDa
PSD95
L'3 L'4 R'1 R'2 R'3 R'4 R'5 W1 W2 W3 W4 75KDa
L'5 L'6 L'7 L'8 L'9 R'6 R'7 R'8 R'9 W5 W6 W7 W8 75KDa
Synapsin-1
L'3 L'4 R'1 R'2 R'3 R'4 R'5 W1 W2 W3 W4 75KDa
75KDa L'5 L'6 L'7 L'8 L'9 R'6 R'7 R'8 R'9 W5 W6 W7 W8
GAPDH/actin
L'3 L'4 R'1 R'2 R'3 R'4 R'5 W1 W2 W3 W4 35KDa
45KDa L'5 L'6 L'7 L'8 L'9 R'6 R'7 R'8 R'9 W5 W6 W7 W8
