# Supplementary material for: Roxadustat (FG-4592) abated lipopolysaccharides-induced depressive-like symptoms via PI3K signaling
Source: Front Mol Neurosci. 2023 Mar 15;16:1048985. doi: 10.3389/fnmol.2023.1048985 (PMC10056220; doi:10.3389/fnmol.2023.1048985)
Supplement: Supplementary file 3 [file Image_1.pdf]

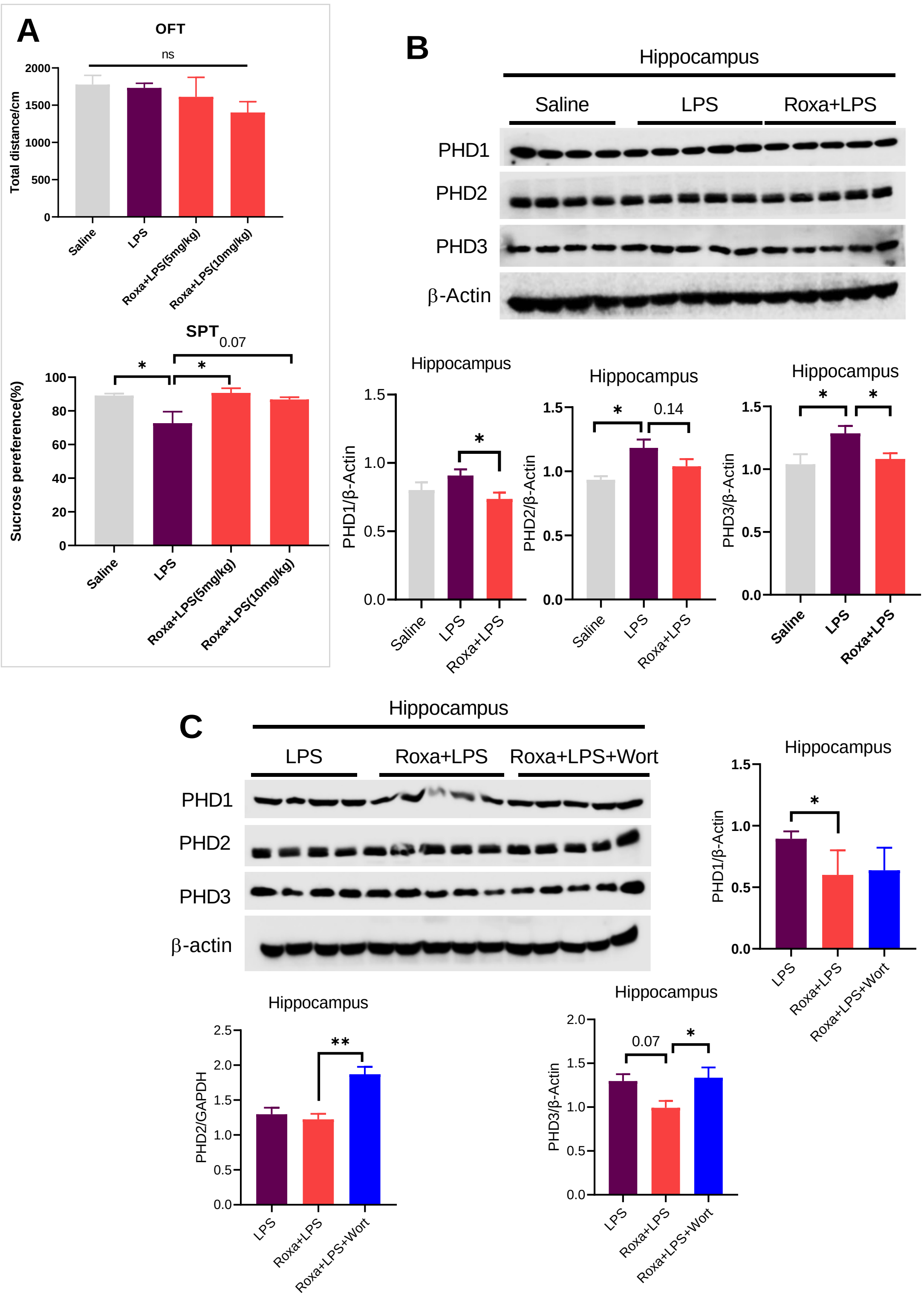
A
OFT
ns
Total distance/cm
2000
1500
1000
500
0
Saline
LPS
Roxa+LPS(5mg/kg)
Roxa+LPS(10mg/kg)
SPT
0.07
*
*
Sucrose pereference(%)
100
80
60
40
20
0
B
Hippocampus
Saline
LPS
Roxa+LPS
PHD1
PHD2
PHD3
β-Actin
Hippocampus
PHD1/β-Actin
PHD2/β-Actin
PHD3/β-Actin
0.14
1.5
1.0
0.5
0.0
C
Roxa+LPS+Wort
β-actin
PHD2/GAPDH
2.5
2.0
**

Figure S1: Roxadustat (5 and 10mg) treatment to mice and PHDs protein expressional changes.

A: Representative bar graphs for OFT and SPT tests when mice were treated with 5mg and 10mg FG4592 in the presence of LPS. **B-C:** Representative immune blots with individual-level column graphs showing expression of PHDs, in the hippocampus of the experimental subjects. All the values were normalized with loading control (β-Actin). Data were expressed as mean ±SEM, One-way ANOVA, and post-hoc analysis. $p = <0.05$ were considered significant. (*): $p <0.05$, (**): $p <0.01$). ns: Non-significant.
